# Inferring the Ancient History of the Translation Machinery and Genetic Code via Recapitulation of Ribosomal Subunit Assembly Orders

**DOI:** 10.1371/journal.pone.0009437

**Published:** 2010-03-01

**Authors:** Gregory P. Fournier, Justin E. Neumann, J. Peter Gogarten

**Affiliations:** 1 Department of Molecular and Cell Biology, University of Connecticut, Storrs, Connecticut, United States of America; 2 Department of Computer Science and Engineering, University of Connecticut, Storrs, Connecticut, United States of America; Johns Hopkins University, United States of America

## Abstract

Universally conserved positions in ribosomal proteins have significant biases in amino acid usage, likely indicating the expansion of the genetic code at the time leading up to the most recent common ancestor(s) (MRCA). Here, we apply this principle to the evolutionary history of the ribosome before the MRCA. It has been proposed that the experimentally determined order of assembly for ribosomal subunits recapitulates their evolutionary chronology. Given this model, we produce a probabilistic evolutionary ordering of the universally conserved small subunit (SSU) and large subunit (LSU) ribosomal proteins. Optimizing the relative ordering of SSU and LSU evolutionary chronologies with respect to minimizing differences in amino acid usage bias, we find strong compositional evidence for a more ancient origin for early LSU proteins. Furthermore, we find that this ordering produces several trends in specific amino acid usages compatible with models of genetic code evolution.

## Introduction

The ribosome is a large complex of RNA and several proteins, composed of two major subunits, the large (LSU) and small (SSU) subunits. Both subunits are highly conserved in all domains of life, and contain several proteins found in every living organism [1], suggesting the core ribonucleoprotein structure evolved before the time of the most recent common ancestor(s) (MRCA). Other ribosomal proteins are domain-specific, evolving at later times within the stem branches of the bacterial and archaeal/eukaryal domains. As the major catalytic peptidyltransferase activity of the ribosome is mediated by RNA, it is postulated that this complex has its origins within the RNA world, evolving from ribozymes with peptide ligase/RNA replicase functions [2]–[4]. As such, the ribosome itself co-evolved with translation and the genetic code, growing in complexity and efficiency as ribosomal proteins were added over time [4]. As the expansion of the genetic code and the stepwise evolution of the “core” ribosome would both have been happening simultaneously up to the time of the MRCA, it is likely that each universal ribosomal protein contains an “imprint” of the genetic code at the time of its recruitment, in the form of biases in amino acid usage at fixed positions. While previous studies have analyzed the overall bias at these positions [5], recapitulating the evolutionary chronology of universal ribosomal proteins from their observed subunit assembly maps [6] allows for a deeper, longitudinal view of changes in amino acid usage. As both the LSU and SSU have independent subunit assembly maps, there exists no *a priori* way to determine the relative ordering of proteins between each chronology; however, assuming that each subunit would be subject to the same pressures of an evolving genetic code, these can be aligned via minimization of the pairwise difference in overall amino acid usage bias observed at each respective chronology position. This results not only in a dataset to examine changes in amino acid usage (and thus genetic code evolution) at times before the MRCA, but also provides empirical evidence for the relative ages of the of the LSU and SSU.

## Methods

### Sequence Retrieval/Alignment

Sequences for ribosomal proteins identified as “universally conserved” [1] were collected from the genbank database (release 164) [7] for all completed archaeal and eukaryotic genomes, and 44 completed bacterial genomes with a wide phylogenetic distribution, totaling 123 genomes. For each ribosomal protein, sequences were aligned within each domain using M-coffee (default parameters) [8]. Aligned protein blocks for each domain were then combined using a profile alignment in ClustalW [9]. Sequences with large deletions at the C or N terminals were omitted from the analysis, as these are possibly due to sequencing or annotation errors, and may interfere with accurate ancestral reconstruction. Neighbor joining trees were also constructed in ClustalW in order to identify genes that may have been subjected to inter-domain transfer; none were found.

Although listed as universally conserved, L16 is lacking in the archaeal and eukaryotic lineages. Earlier analyses have most likely identified L16 as universal due to a low sequence similarity to the archaeal L10e protein; however, the bacterial L10 protein shows much stronger sequence similarity to L10e, and L16 is likely a bacterial-specific ribosomal protein resulting from a duplication and divergence of L10. This is further supported by the terminal location of L16 in the 50s assembly map, as no other universally conserved proteins depend on it for binding.

Additionally, L15 is not found to have homologs in either the archaeal or eukaryal domains. While there is a protein annotated as L15 in archaea and eukarya, it is not homologous. Unlike L16, however, L15 is apparently required for proper universal ribosomal assembly, contributing to the binding of universal proteins L18, L6, and L10. This could be due to a nonorthologous displacement of L15 by another protein in archaea/eukarya, assuming L15 is an ancient protein, or a modification of the ribosomal assembly machinery to include a bacterial-specific L15 protein. For the purposes of analyzing the binding order dependencies, it was assumed that L15 is still actually part of the universal set; however, it was not included in any of the weighted averages, as it is impossible to infer universally conserved positions using representatives from only one domain.

L11 was identified in both bacteria and archaea, although a eukaryal homolog was not identified. The protein annotated as “L11” in eukaryotes is homologous to the L5 ribosomal protein in bacteria and archaea. This dataset was still utilized, however, as the root of the archaea (between the crenarchaeotes and euryarchaeotes) contains a sufficient number of domain-specific positions to contribute to the analysis. The high sequence similarity found between other archaeal and eukaryal ribosomal proteins suggests that the impact on positions determined to be universally conserved is minimally impacted by omission of the eukaryotes.

In the SSU, several subunits identified as universally conserved are dependent on bacterial ribosomal proteins S16, S6, and S18 for binding [10]. However, these proteins seem to be absent in Archaea and Eukarya, likely caused by a non-orthologous gene displacement [11]. Additionally, S20, which contributes to the binding of S16, was found only in the Bacteria, and may also have been displaced, or lost due to the displacement of S16. For this reason, S16, S6, S18, and S20, while included in the assembly map, were not used in calculating weighted means for amino acid usage in the final analysis. SSU protein S21 also was not found in Archaea or Eukarya; however, since this is a terminal protein in the assembly, it was omitted from the assembly map and is not considered in this analysis.

### Tree and Ancestral Sequence Reconstruction

For each ribosomal protein, domain-specific maximum likelihood trees were generated for both bacterial and archaeal/eukaryal sequences, using PHYML (JTT substitution model, 4 rate categories, estimated α, estimated gamma distribution parameter) [12]. These trees were then used in conjunction with ANCESCON (O-option and no optimization of Π–vector) [13], to generate probabilistic sequence reconstructions at ancestral nodes.

### Analysis of Ancestral Sequences

For each bacterial and archaeal/eukaryal ribosomal protein tree, the ancestral sequence corresponding to the root was identified. For archaeal/eukaryal trees, the branch separating domains was identified as containing the root. The biological root of the bacterial domain is not as well characterized. For these trees, in each case a few deep branches were identified that may contain the root, with ANCESCON's midpoint rooting function invariably agreeing with one of these selections. The specific location of the root is irrelevant for this analysis, as the nodes between the very short branches deep in the tree correspond to highly similar sequences, which only differ at positions which were not used based on the stringent ancestral probability criteria described below.

### Ancestral Amino Acid Usage

For bacterial and archaeal/eukaryal root reconstructions, amino acid positions were identified as “conserved” if the probability of their ancestral identity in a given position at both nodes flanking the branch containing the root were each at least 90%. In this way, a high level of confidence in amino acid identities at identified conserved positions is maintained, while permitting occasional derived substitutions along terminal branches. Perfect conservation (i.e., 100% confidence in ancestral position identity), would result in a large reduction in dataset size due to the “false negative” exclusion of many positions due to the presence of these occasional derived states. Predicted ancestral sequences for both bacterial and archaeal/eukaryal datasets were then aligned using M-coffee. Aligned positions designated “conserved” within both bacterial and archaeal/eukaryal analyses were identified as “universally conserved”. Universally conserved positions (*U*) were subsequently subtracted from the bacterial and archaeal/eukaryal datasets of conserved positions, in order to generate “bacterial-specific” and “archaeal/eukaryal-specific” datasets. Combined, these comprise the “domain-specific” dataset (*D*) of positions conserved at the root of either domain, but not within the common ancestor. Amino acid usages were then normalized into rates (% of conserved positions per protein for *U* and *D*, respectively) (Table S1).

### Assembly Maps

**Figure 1:**
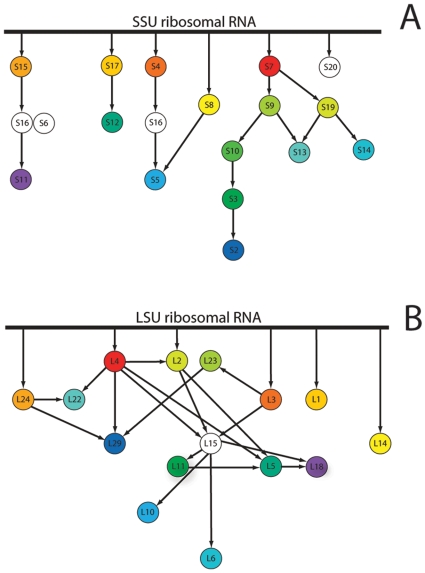
Binding dependencies in subunit assembly used as a rule set for inferring ribosomal evolutionary history. Arrows depict direct dependencies for binding of specific proteins to either the SSU (A) or LSU (B) assembly complex. Arrows from the top line indicate direct binding to rRNA not dependant on other proteins. Colors are protein-specific for each subunit, and match those used in Figure 2. Proteins colored white were included in the assembly rule sets, but omitted from subsequent analyses as described in the text.

The LSU assembly map was adapted from [6] (Figure 1A). The SSU assembly map was adapted from [10] (Figure 1B). Binding dependencies were restricted to a computationally manageable size by removing weaker dependencies that are redundant in determining binding order. Universal proteins that likely underwent non-homologous displacement were included in generating the initial binding order probabilities, then removed from the analysis, with the remaining probabilities re-calculated (effectively giving them a weight of zero in AA usage). Binding dependencies were then listed as “rules” that constrain a set of permitted linear evolutionary orders (PLEOs).

### PLEO Algorithm

**Figure 2:**
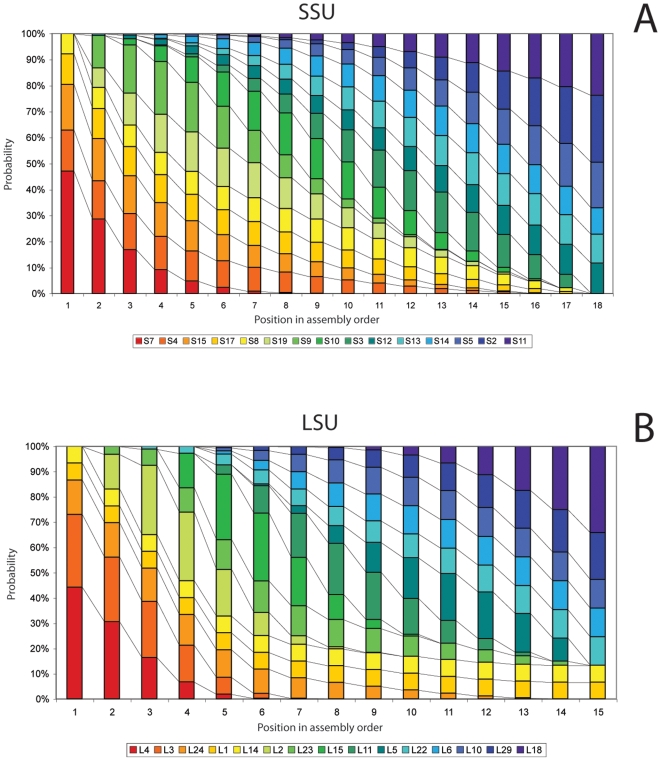
Protein assembly order probabilities based on binding dependencies. Probabilities of each protein being at each position in a linear binding order were determined by compiling an exhaustive exploration of all 30,298,800 permitted linear evolutionary orders (PLEOs) for the LSU (B), and a random sampling of 100,000 PLEOs for the SSU (A). These probabilities are used as weights for each subunit's contribution to the amino acid composition at each position in the linear chronology. Colors are protein-specific for each subunit, and match those used in Figure 1.

An algorithm was devised for exploring all possible PLEOs for the LSU. Of the approximately 1.3×10^13^ possible linear arrangements of LSU proteins, 30,298,800 (0.002%) were found to be permitted given the constructed rule set. Since the SSU rule set is less restrictive, an exhaustive search of PLEOs could not be performed. Therefore, a heuristic version of the algorithm was used, to compile 100,000 randomly generated PLEOs. Using the LSU rule set as a test case, it was determined that this level of sampling would result in a 97.5% accurate result. Furthermore this result was over 95% similar to results for an SSU rule set sampling of 50,000 random PLEOs, suggesting the sampling curve has flattened by this point. Results were compiled per protein, producing a matrix which indicates the probability of each subunit protein being used at any given position in the linear ordering of LSU or SSU proteins, corresponding to its relative position in evolutionary time, the “ribosomal evolutionary order” or REO (Figure 2). The program to implement PLEO algorithms (both exhaustive and heuristic) is available as Source Code S1.

### Weighted Usages

The weighted mean difference in the usage rate of each amino acid (A) for each order position in the REO (*i*) was calculated given the probability (*P*) of each protein (*j*) at *i*. The difference in counts for each given amino acid at conserved positions in *j* (*U_jA_−D_jA_*) was normalized by dividing by the difference in overall amino acid counts (*T*) in *j* (*U_jT_ −D_jT_*):
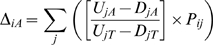



### Chronology Mapping

**Figure 3:**
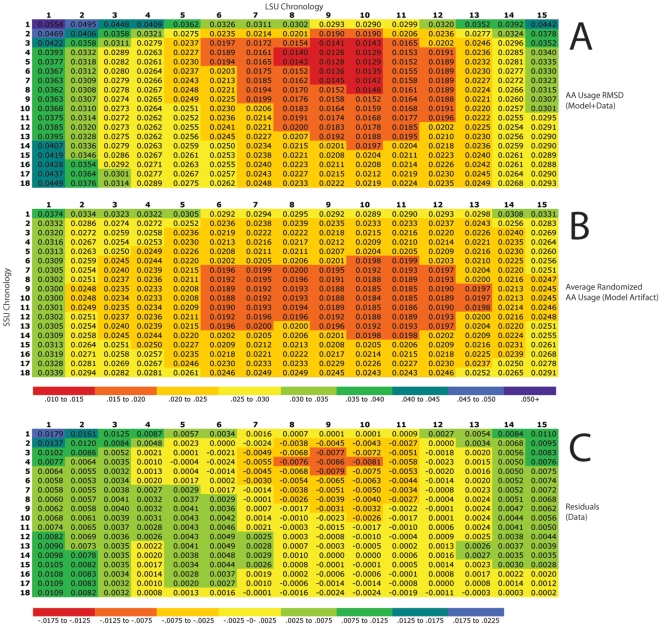
RMSD matrices of composite AA usage similarity between all pairwise positions in LSU and SSU chronologies. Lower RMSD scores indicate a closer match between overall AA usage between LSU and SSU at specific positions in their respective chronologies. Chronology positions correspond to ribosomal evolutionary order (REO) positions (*i*) for both the LSU and SSU. Subtracting a matrix of average RMSD values generated from randomized AA usage data (B) from raw RMSD scores (A), a matrix of residuals is generated (C), free of potential artifacts imposed by the model, as described in the text. Color-coding scales for (A) and (B) differ from that of (C), as by definition RMSD values can only be positive, as opposed to their associated residuals.

Weighted mean differences in overall amino acid usage between each position in the LSU and SSU chronologies (*i_L_*, *i_S_*) were compared using a measure of root mean square distance (RMSD) between their respective relative usage levels for all n = 20 amino acids:
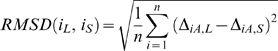
These RMSD values were then used to populate a matrix with (*i_L_*, *i_S_*) coordinates (Figure 3A).

### Removing Model Artifacts

The RMSD matrix is derived from a model where sites are not independent (i.e., positions in the chronologies are weighted averages using proteins often also present in adjacent positions) and with unequal variances across chronologies (early and late chronology positions are weighted averages generated from fewer proteins, and therefore show greater variance). In order to remove these possible sources of bias, a “background” RMSD was generated, averaging 10,000 RMSD matrices produced using D*_iA_* values generated from *U* and *D* values randomized across proteins (Figure 3B). Subtracting the resulting background matrix from the RMSD matrix of the actual data produces the residual matrix (Figure 3C) on which the chronological fitting algorithm was performed.

### Comparison with Randomized Matrices

In order to determine if the RMSD residual matrix contains statistically meaningful structure, it was compared with the residuals of RMSD matrices generated from randomized AA usage data (see previous section). If the original data is structured, then the randomization procedure should impact RMSD matrix values by causing a decrease in both the variance and average RMSD score of the matrix, as the differences between weighted average amino acid usages will become more “flat”, and therefore more similar between chronologies at more positions. This was in fact observed, as randomized residual RMSD matrices showed considerably less variance (1.68×10^−5^ vs. 1.91×10^−5^, one-sample Z test, one-tailed *p* = 0.0334) and much lower average RMSD values (0.00028 vs. 0.00203, one sample Z test, one-tailed *p*<0.0001).

### Optimal Chronological Fitting

A distance-minimizing algorithm was developed to identify the optimal temporal relationship between LSU and SSU chronologies. While a dynamic programming approach, similar to an optimal sequence alignment algorithm such as Needleman-Wunsch, could be used in this case, such an approach would fail to determine the robustness of the revealed optimal path (i.e., how closely similarly-scoring paths match the optimal path), or how much different path regions contribute to the optimal score, given variation in path-density. 10 million random walks through the pairwise RMSD residual matrix were performed, identifying the average RMSD value between LSU and SSU datasets for each walk. This number of iterations was likely to find an optimal value, as several independent runs converged on the same path solution. The rules for each walk are as follows: Given a matrix with dimensions *a_m,n_*, all walks must begin on a randomly selected position of either *a_0,j_* or *a_i,0_*. All walks must end on a position of either *a_i,n_* or *a_m,j_*. In between, any position of *a_i,j_* can be followed by either *a_i+1,j_*, *a_i,j+1_*, or *a_i+1,j+1_*, provided the preceding position was not *a_i−1,j_* in the case of *a_i,j+1_*, or *a_i,j−1_* in the case of *a_i+1,j_*. This maintains the exclusivity of all pairwise positions in a given path.

### Determination of Probable Path-Space

**Figure 4:**
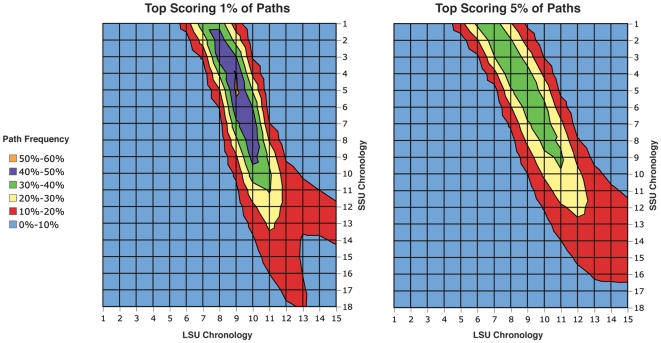
Best-scoring path frequencies based on RMSD residual matrix. Regions with high path frequency indicate a high probability of being included in an optimal pairwise alignment of LSU and SSU chronologies. The best-scoring paths are most congruent with a later and more rapid emergence of protein subunits comprising the SSU.

Scores of walks were compiled into a histogram, with the 5% and 1% best-scoring walks (lowest average RMSD scores) mapped onto a matrices of path-space reflecting the frequency of each position being present in a path, generating a probability landscape relating the chronologies of the LSU and SSU subunits (Figure 4).

Based on this model, top-scoring path landscapes strongly support a more ancient origin of the earliest LSU proteins in chronology positions 1–6 (L4, L3, L2), with the earliest SSU proteins only being added later. From this point, the model supports SSU proteins being added at a faster rate until about SSU position 11/LSU position 11. After this point path space flattens considerably, with the relative ordering of SSU 11–18/LSU 11–15 being indeterminate. Residuals of RMSD matrices generated from randomized data generally produced probability landscapes with significantly lower densities and/or stronger diagonal trends, suggesting that this path is not the result of a persistent artifact.

### Specific Amino Acid Trends

The path landscape model generated from RMSD values should also be reflected in congruent trends in individual AA usages across the aligned SSU and LSU chronologies (Figure S1). Furthermore, there should be a consistent trend of convergence toward the “expected” (*U_c_*−*D_c_*) values for each amino acid across the aligned chronologies. The following test for congruence was performed for each amino acid, using a pairwise alignment of SSU and LSU chronology positions approximating the path landscape model. For each aligned chronology position *c*, D and its corresponding weighted standard error (SE) for SSU and LSU values were used to calculate a Z-score (*Z*) using a two-sample Z test:
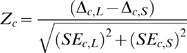
These scores for all aligned positions *k* were then combined into an overall significance score for the congruence of the aligned chronologies using a weighted Z-transform test:
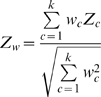
with weights for each position (*w*) given by the inverse combined error variance:
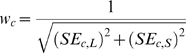
Significance of Z_w_ was then calculated as a two-tailed probability (*p*) (Table 1).

**Table 1:** Congruence and convergence across chronology alignment for specific amino acid usages.

AA	Congruence	Congruence	Convergence
	*p*(Z*_w_*)	(positions)	*Z_V_* (LSU, SSU)
Ala	0.105	15/16	0.667, 1.010
Cys	0.002	14/16	−0.795, −1.120
Asp	0.453	16/16	−1.803, −1.212*
Glu	0.018	16/16	0.436, 4.340
Phe	<0.0001	10/16	1.844, −4.539
Gly	0.006	9/16	−10.338, 2.510
His	<0.001	7/16	−0.101, −0.770
Ile	0.352	14/16	−1.281, 0.131
Lys	0.254	16/16	1.666, 1.652
Leu	0.603	16/16	−0.406, 1.024
Met	0.116	16/16	0.754, 1.821
Asn	<0.001	0/16	−0.675, −2.095*
Pro	0.001	12/16	0.943, −0.845
Gln	<0.001	10/16	−0.212, −2.352*
Arg	0.139	14/16	2.124, −0.495
Ser	0.289	16/16	−1.433, 3.051
Thr	0.020	14/16	0.232, −0.310
Val	0.289	16/16	0.549, −0.736
Trp	<0.001	0/16	0.113, −3.828*
Tyr	0.089	16/16	−5.160, 0.559*

Positions in chronology alignment are congruent between SSU and LSU if *Z_c_*<1.282 (*p*>0.10). Chronologies are convergent if *Z_V_*<−1.000, and divergent if *Z_V_*>1.000.

*Consistently convergent (at least one chronology with *Z_V_*<−1.000, neither with *Z_V_*>1.000, and *Z_V(ave)_*<−1.000.)

In general, trends for individual amino acids show a much weaker signal of congruence than the composite signal for the chronology alignment. The strongest congruence is seen for Asp, Ile, Lys, Leu, Met, Arg, Ser, Val, and Tyr, all of which have significant (*p*<0.05) overall Z*_w_* values as well as congruence in at least 14 out of the 16 positions in the chronology alignment (Table 1). Additionally, Ala shows moderate congruence (p = 0.105, 15/16 positions). Of these congruent signals, Ile, Leu, Asp, and Arg all have a composite signal of increase in relative usage over the chronology, while Lys and Val show a composite signal of decrease in relative usage. Due to the high variance resulting from a small sample size, no trend can be determined for Met. Interestingly, SSU and LSU chronologies show congruence for a relative increase in Ser usage until about LSU positions 8–10/SSU positions 2–4, followed by a relative decrease. Ala usage shows a consistent increase across the LSU chronology, which is congruent with an increase following SSU position 6. Before SSU position 6 Ala usage is much higher, due to an abnormally high level of Ala in ribosomal protein S7, the only ribosomal protein containing dramatically more Ala than Gly at conserved positions. Since S7 is weighted heavily at early positions in the SSU chronology, omitting it results in a much higher overall congruence, supporting a relative increase in Ala usage over time. Tyr showed a consistent and flat under-representation across the chronology alignment.

Of amino acids which failed to show significant congruence across the aligned LSU and SSU chronologies (Cys, Glu, Phe, Gly, His, Asn, Pro, Gln, Thr, Trp), several still showed consistent under-representation across the entire chronology alignment (Trp, Phe, Glu). Due to high variance resulting from small sample sizes, little can be determined from the trends for His or Cys. Both Gln and Asn showed relatively flat usages at expected levels across the LSU chronologies, with correspondingly flat under-representations across SSU chronologies. Relative usage of Thr decreases across the LSU chronology, from an over-representation of 6% to an under-representation of 3%. As usage across the SSU chronology remains flat at expected levels, the aligned chronologies fail the test for overall congruence while showing individual congruence at 14/16 of aligned positions, suggesting a composite signal of decreasing Thr usage. Similar to Ser, Pro usage increases across the LSU chronology until positions 7–9, then decreases. However, Pro is consistently over-represented in the SSU chronology, showing significant disagreement from LSU position 17 on. Of all amino acids analyzed, Gly shows both the strongest and most disparate signal, with a strong decrease in relative usage across the LSU chronology, contrasted with a strong increase in relative usage across the SSU chronology. However, Gly is over-represented at all positions across both chronologies, showing the most over-representation of any amino acid.

### Convergence Trends

Comparing the first and last positions across both aligned chronologies for each amino acid, convergence toward expected values (*U_c_*−*D_c_* = 0) is measured as *Z_V_*, the absolute change in the difference in the number of SE from the expected value:
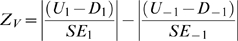
For both chronologies, there was an average slight convergence across all amino acid usages (*Z_V,L(ave)_* = −0.644, *Z_V,S(ave)_* = −0.110), with five amino acids showing consistent significant convergence (Asp, Asn, Gln, Trp, Tyr), three showing consistent significant divergence (Glu, Lys, Met), and four with significant conflicts between *Z_V,L_* and *Z_V,S_* (Phe, Gly, Arg, Ser). The remainder show flat trends with no consistent convergence or divergence (Table 1). Across both chronologies, 4 of the 5 strongest trends were towards convergence (*Z_V,L(Gly)_* = −10.34, *Z_V,L(Tyr)_* = −5.16, *Z_V,S(Phe)_* = −4.54, *Z_V,S(Trp)_* = −3.83). Counterintuitively, some amino acids which show divergence or convergence in average relative usage across the chronology actually have *Z_V_* values indicative of the opposite trend, as error often varies substantially across the alignment (e.g., *U_1,L(Tyr)_−U_−1,L(Tyr)_* vs. *Z_V,L(Tyr)_*).

## Results and Discussion

According to this model, all amino acids that show convergence are in the process of increasing in relative usage (Asp, Tyr, Asn, Gln, Trp), suggesting they were more recently added to the genetic code. Of these, Tyr and Trp still show the greatest under-representation at most recent positions, suggesting these were the latest additions, in agreement with biochemical, metabolic, and phylogenetic arguments [14]. Additionally, Ile, Val, Ala, and Leu show both significant congruence and usage levels near expected values across chronologies. Interestingly, these comprise the complete set of hydrophobic, non-aromatic amino acids. While not statistically significant across the entire chronology, there appear to be local trends showing a relative decrease in Val_S_ corresponding to an increase in Ile_L_ and Leu_L_, which may indicate Val as the precursor aliphatic amino acid, in agreement with models of code evolution based on abiogenic precursors and metabolic complexity [14].

Presumably, Asp/Asn and Glu/Gln represent co-evolved amino acids, as in both cases the latter is still often synthesized from the former via a tRNA-dependent transamidation pathway [15]–[17]. While Glu shows both divergence and fails the test for overall congruence (although showing individual congruence at 16/16 positions), it is also consistently under-represented across both LSU and SSU chronologies, suggesting it is a more recent addition to the code than Asp. No such distinction is discernable between Gln and Asn, which show highly similar trends to one another, specifically in convergence for the SSU dataset.

Phe and Gly both show little congruence between LSU and SSU chronologies, as well as conflicting signals for convergence, with Gly*_L_* and Phe*_S_* showing significant convergence, and Gly*_S_* and Phe*_L_* showing significant divergence. While in both cases datasets showing convergence have much stronger trends than those showing divergence, a simple explanation for this disparity is not readily available. Similarly, usage levels for Cys, Met, and His are too low and sporadic for any meaningful inferences to be made about their role in genetic code evolution, aside from the absence of any consistent under- or over-representation, implying they are unlikely among the first or the last added to the code.

As Ser and Thr are physiochemically similar and often substitute for one another, differences in their relative usage over time are unlikely to be explained by differences in their structural or functional roles within proteins along each chronology. While both are typically considered to be “early” amino acids for various reasons [14], the under-representation of Ser at early positions in each chronology suggest rather that Thr predated Ser. The transient rise observed in Ser is also absent in any other amino acid (except Pro, which, unlike Ser, shows over-representation for most positions, as well as a marked lack of congruence between subunit chronologies).

One alternative hypothesis for explaining some of these observed trends is that earlier protein additions to the ribosomal machinery would have more conserved positions involved in specific RNA-protein interaction, while later additions would have more conserved positions involved in specific protein-protein interaction. This would predict that proteins deeper in the assembly order (and therefore earlier in the chronology) would be enriched at conserved positions in amino acids with higher RNA-protein interface propensities such as positively charged residues that interact with the phosphodiester backbone (Lys, Arg) as well as other amino acids which can be involved in stacking interactions with RNA bases (His, Trp, Tyr). Additionally, Gly is frequently favored in positions adjacent to RNA-binding residues due to its conformational flexibility. Conversely, negatively charged amino (Asp, Glu) and hydrophobic (Ile, Leu, Val, Phe, Ala) amino acids are avoided [18].

Evaluating the validity of this alternative hypothesis is difficult, as the presence and extent of a bias induced by these criteria is impossible to determine without a means of independent comparison. Nevertheless, it could possibly explain the otherwise puzzling congruent (yet divergent) usage of Lys in both chronologies, as this amino acid should be the most strongly correlated with RNA-binding contacts. In this scenario, Lys would be a newer amino acid, with Arg (or a predecessor, possibly ornithine or citrulline) [19], [20] a more ancestral RNA-binding residue. As a later addition to the code, Lys would be under-represented at conserved positions, as has previously been reported [5]. However, if newly-available Lys consistently conferred an advantage by replacing Arg at some RNA-binding positions, it would become enriched at earlier positions within each chronology, resulting in overall usage more closely resembling the expected, and matching the observed trends. The strongly conflicting trends observed between the LSU and SSU for early usage of Gly and Phe would argue against this scenario, however, as one would expect the effect to consistently cause an over-representation in the former, and an under-representation in the latter. However, it is possible that the bias in amino acids other than Lys (and possibly Arg) at early RNA-binding positions is too weak to be detected in these analyses [18].

Another source of bias could be the independent origin of each subunit in a distinct biological environment. While the existence of genetically-encoded proteins presumes a functioning translation machinery, it is possible that protein recruitment to a purely RNA-based “proto-ribosome” could have occurred for the LSU and SSU within different lineages (or sets of lineages) with distinct genetic codes, and therefore reflect distinct code histories. A similar model of code evolution has been proposed to explain the partitioning of aminoacyl-tRNA synthetases into two distinct, non-homologous classes [21]. Using this model, amino acids which show congruence across the histories for the LSU and SSU would simply be those that were shared between the codes of these two groups, while amino acids showing noncongruence and divergence may have been exclusive to one or the other, or possibly in competition with encoded amino acids that did not make it into the “universal” genetic code. This could explain the divergent usages seen for Gly and Phe, as well as the unusual pattern of usage for Ser. The problem with this model is that eventually some coalescence via HGT or other type of fusion must take place. It is difficult to see how this could occur if each biological system had a different code and could not correctly translate and incorporate the proteins of the other. One possible outcome may be subsequent “repartitioning” of coding space, which may be apparent in the unique arrangement of the codons representing Ser in the modern genetic code of most organisms.

The overall trends of amino acid usage across the assembly maps of the LSU and SSU are most congruent with an evolutionary history in which the initial protein component of the LSU predated that of the SSU. Applying the optimized alignment of subunit chronologies using this model to individual amino acid usages, several (albeit weaker) trends in congruence and convergence are observed which are in agreement with certain models of genetic code evolution. Specifically, this model provides additional support that Tyr, Trp, and Glu were among the more recent additions to the genetic code, along with possibly Asp, Lys, Ile, and Leu. While less clear support exists for identifying more ancient amino acids, this set most likely consists of Gly, Thr, Pro, and possibly Ala and Val.

## Supporting Information

Figure S1 Trends in usage of specific amino acids across aligned subunit chronologies. Grey regions indicate aligned positions between LSU and SSU chronologies given the model depicted in Figure 4. Aligned positions are numbered with respect to LSU chronology. Repeat numbers followed by parentheses indicate positions where more than one consecutive SSU position correlates to the same chronological position within the LSU. Corresponding graphs use different scales of Δ for clarity. Note that the c-axis is ordinal and does not correspond to regular time intervals.
(0.33 MB XLS)

Source Code S1 Programs for the exhaustive and heuristic algorithm to calculate permitted linear evolutionary orders (PLEOs). This folder contains the executable program, readme file, and sample dataset/output files. The source code is Gnu GPL licensed. The folder is a .tar.gz zipped file, and requires use of tar -xvf after gunzip.
(0.23 MB GZ)

Table S1 Amino acid usages at conserved positions for universal ribosomal proteins. Raw counts and percentages are included for universal (U) and domain-specific (D) positions for both LSU and SSU proteins.
(0.07 MB XLS)
