# Supplementary figures and images for: Inferring the Ancient History of the Translation Machinery and Genetic Code via Recapitulation of Ribosomal Subunit Assembly Orders
Source: PLoS One. 2010 Mar 1;5(3):e9437. doi: 10.1371/journal.pone.0009437 (PMC2830423; doi:10.1371/journal.pone.0009437)

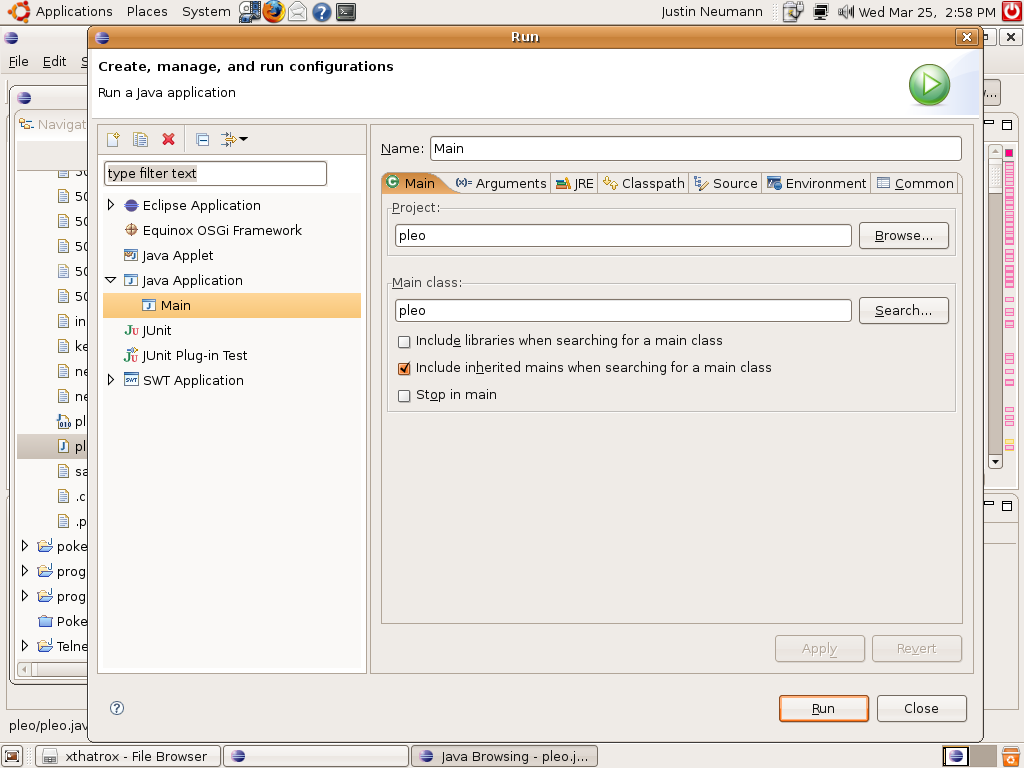

Supplement: Source Code S1 — Programs for the exhaustive and heuristic algorithm to calculate permitted linear evolutionary orders (PLEOs). This folder contains the executable program, readme file, and sample dataset/output files. The source code is Gnu GPL licensed. The folder is a .tar.gz zipped file, and requires use of tar -xvf after gunzip. (0.23 MB GZ) [file pone.0009437.s002.gz › pleo/pleo_run.png]

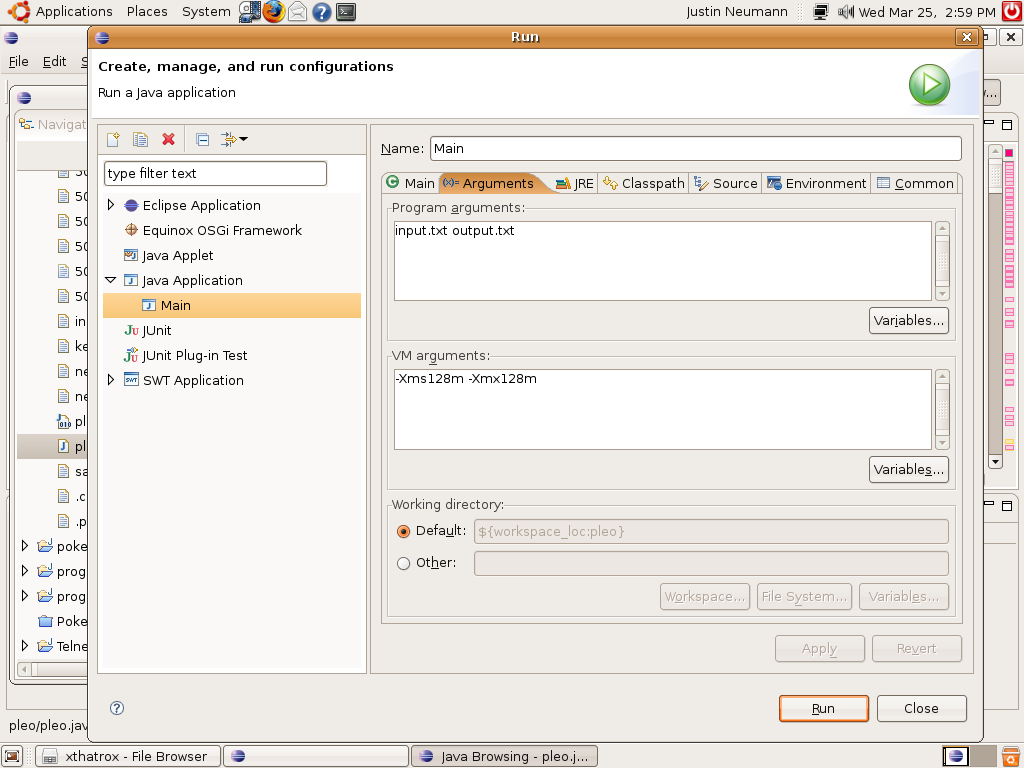

Supplement: Source Code S1 — Programs for the exhaustive and heuristic algorithm to calculate permitted linear evolutionary orders (PLEOs). This folder contains the executable program, readme file, and sample dataset/output files. The source code is Gnu GPL licensed. The folder is a .tar.gz zipped file, and requires use of tar -xvf after gunzip. (0.23 MB GZ) [file pone.0009437.s002.gz › pleo/pleo_arguments.png]
